# Treatment of Eczema

**Published:** 1875-08

**Authors:** 


### TREATMENT OF ECZEMA.

Dr. L. D. Buckley, of New York, in an interesting "Analysis of 1000 Cases of Skin Disease" (*American Practitioner*, May, 1875), gives the following resume of his treatment of eczema:—

"I do not order poultices to remove the crusts of infantile eczema, as many do, preferring much to cause their separation by means of fatty matter. Among the poor, and sometimes among the rich, I have the head soaked in cod-liver oil (sweet-almond oil answers), or I have the ointment appled at once in a tolerably s ft form; directing that the head shall not be washed at all, but as fast as the crusts fall, perhaps with slight assistance from the finger-nail, the ointment is to be re-applied; the idea being to thoroughly protect the irritated mucons layer of the skin, and to shield it from air and water. Occasionally the crusts will accumulate and adhere, and it becomes necessary to use a poultice or wash the head well with warm water and borax; but this, in my experienee, is very rare.

"During the past year I have empolyed very largely tannin in ointment (one drachm to one ounce) in eczema, and like it very well. A very common treatment is to bathe first with the *liquor picis alkalinus*, diluted ten or twelve times, twice a day, and apply the tannin ointment immediately afterward. I have also used with very satisfactory results the subnitrate bismuth in ointment (half a drachm to one ounce), and perfer it in very many instances to that of zinc, as commonly employed. I would again mention the value of the rose ointment as an excipient, and its efficiency when the simple ointment has failed. Several cases of eczema rubrum, covering quite a large part of the body of children one or two years old, were seen. These case are often most obstinate. Our best results were attained by starch and alkaline baths, and powdering the surface with subnitrate of bismuth and starch.

"Internal treatment is alwasys required, and I believe that the largest percentage of good results was obtained by means of cod-liver oil in appropriate doses. Syrup of the iodide of iron is also invaluable in treating eczema in children.

"In adults most of the cases of eczema were of the chronic form, very many of them being in the legs, and dependent upon varicose veins. The treatment of these is very frequently unsatisfactory, because of the continued existence of the cause, especially among the poor, who cannot give the necessary time to rest. Elastic stockings should be insisted on the eczema of the legs when the disease has recurred often or lasted long; for, although the veins may not appear to be varicose, there is often a want of tone of the capillaries, which is supplied by the stockings. We have had good results from the use of tarry preparations, and I [illegible]ave known a moist eczema to be completely healed after a very few applications of the *liquor picis alkalinus* in full strength. A common treatment in chronic eczema is equal parts of tar and oxide-of-zinc ointments, with the addition of a little mercurial ointment, as the citrine, when the surface ceases to be moist.

"In place of the *sapo viridis*, or green potash soap of the Germans, I have been employing the ordinary American soft soap made with potash, and with almost, if not quite, as good results, although it contains relatively less potassa. In one case of eczema of the hands, in a mason aged thrty-three years, which had existed for ten or more years, it was used with excellent effect. He had been treated by me with other measures for six months with varying success, and when this method was commenced the skin on the back of both hands was very greatly thickened, even to three or four times the normal; the surface was hard and scaly in some places, moist and cracking in others. He was first given a strong potash solution (one drachm to one ounce), with which the surface was well rubbed once or twice, and covered with the diachylon ointment of the Germans. This caused great swelling, which subsided, leaving the parts somewhat less thickened. He was then directed to rub in the common soft-soap well, night and morning, and cover the hands as before; and after a short time the friction with which it was applied was increased, until he came to using an ordinary scrubbing-brush, such as is used for the floor. Dipping it in soft-soap, the back of each hand was scrubbed—the palm resting on a table, till the opposite arm and shoulder were tired. The results was that at each visit a marked diminution in the thickness was noticed, and in three weeks the skin was reduced to almost the normal thickness, and his hands were better than they had been for ten years. This is an exaggerated case, but is of value, showing how far the stimulating treatment may be pushed with advantage; whereas, on the contrary, nintey out of one hundred of the ordinary run of eczema cases would be greatly aggravated by such means.

"In one case of eczema of the scrotum I obtained very excellent results from the repeated application, by means of a camel's-hair brush, of the compound tincture of benzoin. The man ceased attending before the thickening had entirely disappeared, and the ultimate results cannot be stated with certainty; but it is probable that the disease was cured, and the remedy was the first one tried by me, and the relief and satisfaction expressed by the patient was very great.

"Quite a large share of the cases of ordinary eczema of various parts was treated by the oxide-of-zinc ointment, very generally in conjunction with some internal medication, depending upon the state of the patient. Many of this class are the constant subjects of dyspepsia, and the rhubarb and soda mixture was very commonly used. I frequently add Fowler's solution to it, giving of the latter three or four drops with a teaspoonful of the former, many of these patients require tonics, and the ammonio-citrate of iron aud compound tincture of cinchona were generally used. Aucte lichenous eczema I frequeutly treated with Startin's mixture of sulphate of magnesia, sulphate of iron, aromatic sulphuric acid, and gentian. Acetate of potassa, alone or combined, was used somewhat, and in my hands has done much for eczema."

